# Research on the Regulatory Mechanism of Algae Reproduction under Abiotic Stress Conditions

**DOI:** 10.3390/plants11040525

**Published:** 2022-02-15

**Authors:** Koji Mikami

**Affiliations:** Department of Integrative Studies of Plant and Animal Production, School of Food Industrial Sciences, Miyagi University, 2-2-1 Hatatate, Taihaku-ku, Sendai 982-0215, Japan; mikamik@myu.ac.jp

The intertidal and subtidal zones are characterized by daily and seasonal fluctuations in environmental conditions. Seaweeds that inhabit these environments face wide-ranging temperatures, nutrient deficiency, changes in salinity, and long periods of desiccation [1,2,3,4,5,6,7]. Accordingly, like terrestrial plants, these seaweeds possess the innate ability to acclimate to environmental stresses [8,9,10,11,12].

The ‘life-cycle trade-off’ is a well-described phenomenon of both algae and terrestrial plants that controls the timing of growth and reproduction in response to environmental stresses; this trade-off can optimize survival by selecting sexual or asexual propagation to promote adaptation to changes in environmental conditions [13,14,15,16]. Despite these similarities, the effects of environmental stresses differ between algae and terrestrial plants. For instance, although heat stress negatively affects reproduction in terrestrial plants [17,18,19], positive effects of heat stress on the sexual life-cycle progression have been observed in sessile red algae of the order Bangiales [13,20] and in the green alga *Volvox carteri* [21]. Thus, elucidation of the regulatory mechanisms of the life-cycle trade-off in seaweeds could provide insights valuable not only for enhancing production during mariculture farming of economically important marine resources but also for sustaining the sea environment via maintenance of seaweed forests.

In ‘*Bangia*’ sp. ESS1 (Bangiales), the asexual life-cycle—which involves the production of asexual spores from thalli—is promoted by heat stress [22]. Moreover, non-lethal temperature stress promotes heat stress tolerance in ‘*Bangia*’ sp. ESS1, which enables survival under otherwise lethal heat stress conditions [23]. Since spore release was observed to coincide with acquisition of tolerance in ‘*Bangia*’ sp. ESS1 [22,23], the promotion of the asexual life-cycle by heat stress is proposed to be triggered by establishment of heat stress tolerance. No spore release was observed in *Neopyropia yezoensis*, a major cultivar of nori in Japan, under heat stress conditions [24]; therefore, it is possible that such promotion of the asexual life-cycle by heat stress and its relationship to the acquisition of heat stress tolerance is genus or species specific in Bangiales and other algae. 

This Special Issue on “Research on the Regulatory Mechanism of Algae Reproduction under Abiotic Stress Conditions” comprises five studies covering aspects of the life-cycle trade-off. They address the effects of the loss of water current on stimulation of asexual life-cycle progression [25], the effects of combined heat and nutritional depletion stresses on promotion of the asexual life-cycle [26], the relationship between heat stress tolerance and loss of life-cycle trade-off ability [27], and the role of a heat stress-insensitive asexual life-cycle trade-off in the maintenance of vegetative growth [28]. In addition, Khoa et al. [29] focus on the intrinsic ability to acquire tolerance to lethal heat stress in different *Bangia* species based on memory of non-lethal heat stress in relation to asexual spore release. Thus, the studies in this Special Issue cover a broad range of recent findings on environmental stress-dependent life-cycle trade-offs in seaweeds. In this Editorial, I summarize the highlights of each study and focus on the promotion of the asexual life-cycle under combined environmental stress conditions.

Omuro et al. [25] explore the acquisition of freezing tolerance and the promotion of the freezing-dependent asexual life-cycle by loss of hydrodynamic stress in ‘*Bangia*’ sp. ESS1. Since Bangiales inhabit the intertidal zone with dynamic water currents, it is likely that hydrodynamic stress is required for their growth and survival. Membrane fatty acids of ‘*Bangia*’ sp. ESS1 were unsaturated during static culture (lacking water current), which resulted in acquisition of freezing tolerance and asexual spore release after thawing. Though the relationship between asexual spore release and unsaturation of membrane fatty acids needs to be elucidated, there is clearly a tight relationship between freezing tolerance acquisition upon loss of hydrodynamic stress and the promotion of the life-cycle trade-off in ‘*Bangia*’ sp. ESS1. 

According to a recent revision of the Bangiales phylogeny, the genus *Bangia sensu lato* was separated into four genera, *Bangia*, ‘*Bangia*’ 1, ‘*Bangia*’ 2, and ‘*Bangia*’ 3 [30]. ‘*Bangia*’ sp. ESS1 belongs to ‘*Bangia*’ 2 [31] and has an intrinsic ability for heat stress memory to acquire heat stress tolerance with the release of asexual spores [22,23]. However, little is known about whether other *Bangia* species also remember and adapt to heat stress. Khoa et al. [29] classified ‘*Bangia*’ sp. ESS2 as ‘*Bangia*’ 3 and compared its heat stress response with those in ‘*Bangia*’ sp. ESS1 and *Bangia atropurpurea* [32]. ‘*Bangia*’ sp. ESS2 was not able to acquire heat stress tolerance and remember previous heat stress, whereas the acquisition of heat stress tolerance but not heat stress memory was observed in *B. atropurpurea*. In addition, the asexual life-cycle was repressed by heat stress in ‘*Bangia*’ sp. ESS2, and *B. atropurpurea* did not release asexual spores under heat stress conditions. Thus, intrinsic heat stress responses, including the life-cycle trade-off, appear to be species-specific. Overall, these findings underscore that there is a relationship between heat stress memory and heat stress-dependent promotion of the asexual life-cycle. 

Endo et al. [26] demonstrate a high tolerance of holdfasts (the equivalent of roots in seaweeds, which anchor the organism to the sea floor) to heat stress in the brown alga *Sargassum fusiforrme*. Under high temperature and low nutrition conditions, holdfasts can grow and regenerate into new shoots by vegetative reproduction, i.e., asexual reproduction. Thus, *S. fusiforrme* proliferates in summer via the regeneration of shoots, suggesting a relationship between high temperature tolerance and transition to the asexual growth phase. In addition, regeneration was enhanced by the fragmentation of holdfasts. Thus, the combined effects of high temperature and nutrition starvation on regeneration could be strengthened by wounding stress. The authors also demonstrate that heat stress tolerance is associated with nitrogen accumulation.

Sato et al. [27] report differences in temperature dependency of growth and sporulation in several strains of the green alga *Ulva prolifera*. Although asexual spore release was generally accelerated at 20 °C in this species, one strain did not sporulate at 20 °C, which is a notable characteristic for mariculture of *U. prolifera* in the face of increases in seawater temperature due to global warming. In addition, although heat stress generally increases nitrogen contents in this species, this strain did not show heat-stress-dependent nitrogen accumulation. Hiraoka [28] support these findings in their study comparing attached-type *U. prolifera* subsp. *prolifera* and bloom-type *Ulva prolifera* subsp. *qingdaoensis*. Although the former produces spores in spring, the latter is fragmented in spring and grows vegetatively in summer, suggesting that sporulation is inhibited under heat and nutrient starvation conditions in the bloom type. These findings indicate that heat stress tolerance is negatively related to the asexual life-cycle trade-off in *U. prolifera* subsp. *qingdaoensis*, which is in contrast to the red alga ‘*Bangia*’ sp. ESS1, although the heat-stress-dependent promotion of vegetative growth in *U. prolifera* subsp. *qingdaoensis* is similar to that in *S. fusiforrme*. These findings again demonstrate that the relationship between stress tolerance and life-cycle trade-off differs among seaweed phyla.

Overall, the studies in this Special Issue increase our understanding of the effects of combined stresses on stress tolerance and life-cycle trade-off, which differ among species, genera, and phyla, and will contribute to the expansion and development of biological research on seaweeds. Elucidation of the mechanisms regulating stress-dependent reproductive responses will enhance our understanding of the flexible life-cycle strategies that enable seaweeds to survive in fluctuating environmental conditions by promoting the life-cycle trade-off. 

## References

[1] Wang W.-J., Zhu J.-Y., Xu P., Xu J.-R., Lin X.-Z., Huang C.-K., Song W.-L., Peng G., Wang G.-C. (2008). Characterization of the life history of *Bangia fuscopurpurea* (Bangiaceae, Rhodophyta) in connection with its cultivation in China. Aquaculture.

[2] Karsten U., West J.A. (2000). Living in the intertidal zone seasonal effects on heterosides and sunscreen compounds in the red alga *Bangia atropurpurea* (Bangiales). J. Exp. Mar. Bio. Ecol..

[3] Zou D., Gao K. (2002). Effects of desiccation and CO_2_ concentrations on emersed photosynthesis in *Porphyra haitanensis* (Bangiales, Rhodophyta), a species farmed in China. Eur. J. Phycol..

[4] Thompson R.C., Norton T.A., Hawkins S.J. (2004). Physical stress and biological control regulate the producer consumer balance in intertidal biofilms. Ecology.

[5] Rawlings T.A. (1999). Adaptations to physical stresses in the intertidal zone: The egg capsules of neogastropod molluscs. Am. Zool..

[6] Helmuth B.S., Hofmann G.E. (2001). Microhabitats, thermal heterogeneity, and patterns of physiological stress in the rocky intertidal zone. Biol. Bull..

[7] Echersley L.K., Scrosati R.A. (2012). Temperature, desiccation, and species performance trends along an intertidal elevation gradient. Curr. Dev. Oceanogr..

[8] Nakashima K., Yamaguchi-Shinozaki K., Shinozaki K. (2014). The transcriptional regulatory network in the drought response and its crosstalk in abiotic stress responses including drought, cold, and heat. Front. Plant Sci..

[9] Rejeb I.B., Pastor V., Mauch-Mani B. (2014). Plant responses to simultaneous biotic and abiotic stress: Molecular mechanisms. Plants.

[10] Zhu J.-K. (2016). Abiotic stress signaling and responses in plants. Cell.

[11] Sade N., Rubio-Wilhelmi M.D.M., Umnajkitikorn K., Blumwald E. (2018). Stress-induced senescence and plant tolerance to abiotic stress. J. Exp. Bot..

[12] Vishwakarma K., Upadhyay N., Kumar N., Yadav G., Singh J., Mishra R.K., Kumar V., Verma R., Upadhyay R.G., Pandey M. (2017). Abscisic acid signaling and abiotic stress tolerance in plants: A review on current knowledge and future prospects. Front. Plant Sci..

[13] Liu X., Bogaert K., Engelen A.H., Leliaert F., Roleda M.Y., De Clerck O. (2017). Seaweed reproductive biology: Environmental and genetic controls. Bot. Mar..

[14] Mohring M.B., Kendrick G.A., Wernberg T., Rule M.J., Vanderklift M.A. (2013). Environmental influences on kelp performance across the reproductive period: An ecological trade-off between gametophyte survival and growth?. PLoS ONE.

[15] Karasov T.L., Chae E., Herman J.J., Bergelson J. (2017). Mechanisms to mitigate the trade-off between growth and defense. Plant Cell.

[16] Shaar-Moshe L., Hayouka R., Roessner U., Peleg Z. (2019). Phenotypic and metabolic plasticity shapes life-history strategies under combinations of abiotic stresses. Plant Direct.

[17] Barnabás B., Jäger K., Fehér A. (2008). The effect of drought and heat stress on reproductive processes in cereals. Plant Cell Environ..

[18] Zinn K.E., Tunc-Ozdemir M., Harper J.F. (2010). Temperature stress and plant sexual reproduction: Uncovering the weakest links. J. Exp. Bot..

[19] Hatfield J.L., Prueger J.H. (2010). Temperature extremes: Effect on plant growth and development. Weather Clim. Extrem..

[20] Kakinuma M., Kaneko I., Coury D.A., Suzuki T., Amano H. (2006). Isolation and identification of gametogenesis-related genes in *Porphyra yezoensis* (Rhodophyta) using subtracted cDNA libraries. J. Appl. Phycol..

[21] Kirk D.L., Kirk M.M. (1986). Heat shock elicits production of sexual inducer in *Volvox*. Science.

[22] Mikami K., Kishimoto I. (2018). Temperature promoting the asexual life cycle program in *Bangia fuscopurpurea* (Bangiales, Rhodophyta) from Esashi in the Hokkaido Island, Japan. Algal Resour..

[23] Kishimoto I., Ariga I., Itabashi Y., Mikami K. (2019). Heat-stress memory is responsible for acquired thermotolerance in *Bangia fuscopurpurea*. J. Phycol..

[24] Suda M., Mikami K. (2020). Reproductive responses to wounding and heat stress in gametophytic thalli of the red alga *Pyropia yezoensis*. Front. Mar. Sci..

[25] Omuro Y., Khoa H.V., Mikami K. (2021). The Absence of hydrodynamic stress promotes acquisition of freezing tolerance and freeze-dependent asexual reproduction in the red alga ‘*Bangia*’ sp. ESS1. Plants.

[26] Endo H., Sugie T., Yonemori Y., Nishikido Y., Moriyama H., Ito R., Okunishi S. (2021). Vegetative reproduction is more advantageous than sexual reproduction in a canopy-forming clonal macroalga under ocean warming accompanied by oligotrophication and intensive herbivory. Plants.

[27] Sato Y., Kinoshita Y., Mogamiya M., Inomata E., Hoshino M., Hiraoka M. (2021). Different growth and sporulation responses to temperature gradient among obligate apomictic strains of *Ulva Prolifera*. Plants.

[28] Hiraoka M. (2021). Massive *Ulva* green tides caused by inhibition of biomass allocation to sporulation. Plants.

[29] Khoa H.V., Kumari P., Uchida H., Murakami A., Shimada S., Mikami K. (2021). Heat-stress responses differ among species from different ‘*Bangia*’ clades of Bangiales (Rhodophyta). Plants.

[30] Sutherland J., Lindstrom S., Nelson W., Brodie J., Lynch M., Hwang M.S., Choi H.G., Miyata M., Kikuchi N., Oliveira M. (2011). A new look at an ancient order: Generic revision of the Bangiales. J. Phycol..

[31] Li C., Irie R., Shimada S., Mikami K. (2019). Requirement of different normalization genes for quantitative gene expression analysis under abiotic stress conditions in ‘*Bangia*’ sp. ESS1. J. Aquat. Res. Mar. Sci..

[32] Yokono M., Uchida H., Suzawa Y., Akiomoto S., Murakami A. (2012). Stabilization and modulation of the phycobilisome by calcium in the calciphilic freshwater red alga *Bangia atropurpurea*. Biochim. Biophys. Acta.

